# Characterisation of the *Drosophila* procollagen lysyl hydroxylase, *dPlod*

**DOI:** 10.1016/j.gep.2010.09.006

**Published:** 2011-01

**Authors:** Stephanie Bunt, Barry Denholm, Helen Skaer

**Affiliations:** Department of Zoology, University of Cambridge, Cambridge, CB2 3EJ, UK

**Keywords:** Drosophila, dPlod, Plod, Collagen, Lysyl hydroxylase, LH3

## Abstract

The lysyl hydroxylase (LH) family of enzymes has important roles in the biosynthesis of collagen. In this paper we present the first description of *Drosophila* LH3 (*dPlod*), the only lysyl hydroxylase encoded in the fly genome. We have characterised in detail the developmental expression patterns of *dPlod* RNA and protein during embryogenesis. Consistent with its predicted function as a collagen-modifying enzyme, we find that *dPlod* is highly expressed in type-IV collagen-producing cells, particularly the haemocytes and fat body. Examination of dPlod subcellular localisation reveals that it is an endoplasmic reticulum resident protein, that partially overlaps with intracellular type-IV collagen. Furthermore, we show that dPlod is required for type-IV collagen secretion from haemocytes and fat body, and thus establish that LH3 enzyme function is conserved across widely separated animal phyla. Our findings, and the new tools we describe, establish the fly as an attractive model in which to study this important collagen biosynthesis enzyme.

Basement membranes are thin mats of specialised extracellular matrix that underlie epithelial sheets and tubes. They provide scaffold support for cell adhesion, cell migration and changes in cell and tissue morphology, they provide interactive surfaces for cell signalling, and they act as selective filtration barriers (Hudson et al., 1993; Hutter et al., 2000; Schneider and Granato, 2006; Vasilyev et al., 2009; Wang et al., 2008). Type-IV collagen is an important and evolutionarily ancient constituent of all basement membranes, forming a flexible, multi-layered extracellular meshwork (Boute et al., 1996; Yurchenco et al., 2004). Macromolecular assembly and secretion of type-IV collagen is dependent upon multiple co- and post-translational modifications. These include processing of lysine residues by the lysyl hydroxylase (LH) enzyme family. Members of this family (also referred to as PLOD or procollagen-lysine, 2-oxoglutarate 5-dioxygenase) catalyze the hydroxylation of lysine residues in collagens and other proteins containing collagenous-like domains. To date, three LH genes (LH1, LH2 and LH3) have been identified in vertebrates and a single LH (LH3) in invertebrates (Myllyharju and Kivirikko, 2004; Myllyla et al., 2007). LH3 is a multifunctional enzyme that, in addition to its LH activity, is capable of catalyzing the glycosylation of hydroxylysyl residues to produce either monosaccharide (galactose) or disaccharide (glucose-galactose) derivatives (Heikkinen et al., 2000; Rautavuoma et al., 2002; Ruotsalainen et al., 2006; Wang et al., 2002a,b). The importance of the LH family for collagen biosynthesis in humans is underlined by a number of connective tissue diseases that are associated with mutations in LH genes. These include Ehlers-Danlos syndrome type VI for LH1 (Steinmann et al., 1993; Yeowell and Walker, 2000), Bruck syndrome for LH2 (Ha-Vinh et al., 2004; van der Slot et al., 2003) and a severe connective tissue disorder whose features overlap with known collagen disorders for LH3 (Salo et al., 2008).

Modification of lysine residues in collagen polypeptides foster the generation of cross-links which are necessary for collagen chain assembly into supramolecular complexes. For example, type IV and type VI collagens are highly decorated by lysine modifications that are important for their assembly into tetramers (Sipila et al., 2007). In the absence of modification collagen secretion is impaired and instead accumulates to high and cytotoxic levels within the cell. In LH3 knock-out mice type-IV collagen secretion fails and the severe basement membrane formation defects that occur as a result lead to embryonic lethality (Rautavuoma et al., 2004; Ruotsalainen et al., 2006). Even a moderate reduction in LH3 activity, in humans or mice deficient for just one LH3 allele, results in defects in deposition and organisation of extracellular matrix (Risteli et al., 2009). The function of LH3 appears to be widely conserved because collagen secretion is also impaired and is absent from basement membranes in LH3 mutant fish (*diwanka* mutants, (Schneider and Granato, 2006) and worms (*let-268* mutants, Norman and Moerman, 2000).

LH3 is widely expressed during mouse embryogenesis but becomes more restricted to specific cells within adult tissues such as brain, lungs, spleen, muscle, pancreas, kidney and liver (Salo et al., 2006a). Immuno-electron microscopy reveals that LH3 localises intracellularly within the endoplasmic reticulum (ER) (Salo et al., 2006a). In addition, LH3 is secreted into the extracellular space surrounding some tissues such as kidney, spleen, muscle and liver (Salo et al., 2006a,b). Studies in cultured cells indicate that LH3 is secreted from cells and located both in the medium and on the cell surface, where it is associated with collagenous proteins (Salo et al., 2006b). These data suggest that collagen modification by LH3 may occur both inside and outside the cell. In early zebrafish embryos LH3 is expressed ubiquitously until gastrulation when it becomes concentrated in the axial mesoderm. It is expressed strongly in the notochord and at low levels throughout the myotome (Schneider and Granato, 2006). In the worm LH3 is detected in the body wall muscles and in glial-like cells where it coincides with the expression of type-IV collagen (Norman and Moerman, 2000).

Here we describe the embryonic expression pattern for *Drosophila* LH3 (*dPlod*), the only lysyl hydroxylase encoded in the fly genome, using a combination of *in situ* hybridisation, antibody staining (using a commercially available LH3 cross-reactive antibody raised against human LH3), and a GAL4 enhancer-trap line. We show that dPlod is strongly expressed in cells and tissues that produce and secrete high levels of type-IV collagen where it partially colocalises with intracellular type-IV collagen in the ER, consistent with its established function in other organisms as a type-IV collagen-modifying enzyme. Finally, using a deficiency that removes the *dPlod* locus we show that dPlod is required for collagen secretion. These data and the novel tools we describe provide a new, genetically tractable model with which to dissect the function and regulation of this important collagen-modifying enzyme.

## 1 Results

### 1.1 *dPlod* is a *Drosophila* lysyl hydroxylase

**Fig. 1:**
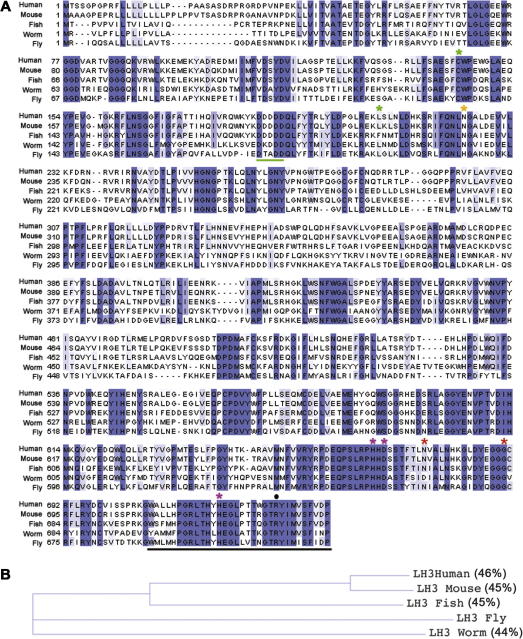
Protein alignment of LH3 proteins across the animal kingdom. (A) ClustalW protein alignment of human, mouse, zebrafish, worm and fly LH3. Shared identical amino acids are indicated by coloured boxes, with dark/light shading representing degree of conservation for each amino acid between the five species. Asterisks above the sequence mark amino acids required for Fe^2+^ binding and LH activity (magenta) as assayed for LH1 and LH3; a disease-causing mutation in a LH3 human patient with reduced GT and GGT activities (position 223, orange); worm mutations in *let-268* (positions 668 and 682, red); residues important for GGT activity in humans and worms (green). Sequence underlined in green corresponds to the DXD motif, a region important for GGT activity. Sequence underlined in black represents the putative ER-retention signal. Black dot represents the 2-oxogluatarate binding site. (B) Cladogram representing relationships between human, mouse, fish, worm and fly LH3. The percentage of amino acid identity is shown (generated by BLASTP).

The fly genome contains a single lysyl hydroxylase gene annotated as CG6199 that we refer to here as *Drosophila* *Plod* or *dPlod*. It maps to cytological position 68B1 on the left arm of the third chromosome and the genomic locus spans 7.9 kb. Two annotated transcripts, CG6199-RA and CG6199-RB, 2660 and 2574 nucleotides in length differ by only 86 nucleotides in their 5′UTR. Conceptual translation of these transcripts yields a 721 amino acid protein with a predicted molecular weight of 82.5 kDa. Protein alignment with LH genes from other species indicates that *dPlod* is more closely related to vertebrate LH3-type and worm LH3 than either of the LH1 or LH2 genes. dPlod possesses on average ∼45% identity and ∼66% similarity with LH3 proteins from humans, mice, fish and worms, with the carboxyl-terminal region having the highest level of conservation (Fig. 1A). A putative ER-retention signal at the extreme C-terminus of dPlod is highly conserved. The phylogenetic relationships between human, mouse, fish, worm and fly LH3 are shown in the cladogram (Fig. 1B).

Several key residues required for the different LH3 enzymatic activities are conserved in dPlod. The carboxyl-terminal region of LH3 proteins corresponds to the lysyl hydroxlase active site. This site, that constitutes (i) the Fe^2+^ binding site including histidine and aspartate residues at positions His656/667, Asp658/669 and His708/719 in human LH1/LH3, respectively (Pirskanen et al., 1996; Ruotsalainen et al., 2006), are conserved in dPlod (amino acid positions 650, 652 and 702, respectively); and, (ii) the arginine residue that binds the C-5 carboxyl group of 2-oxoglutarate (a co-substrate required for hydroxylation activity) (Passoja et al., 1998) is conserved in dPlod (positions 729 and 712 in human and fly LH3). The sites of glycosyltransferase activity have been mapped to the amino-terminal region of vertebrate LH3. In human LH3, site-directed mutagenesis of cysteine 144 and leucine 208 leads to significant reduction in glycosyltransferase activity when tested *in vitro* (Wang et al., 2002a,b). These residues are conserved in dPlod (Cys133 and Leu197). In addition, three out of five aspartates in the so-called D-rich region, which is also indispensable for glycosyltransferase activities, are conserved in dPlod (Wang et al., 2002a,b). Our *in silico* analyses strongly suggest that fly dPlod possesses lysyl hydroxlase and glycosyltransferase activities demonstrated previously for LH3 enzymes from other species.

### 1.2 Embryonic expression of *dPlod*

**Fig. 2:**
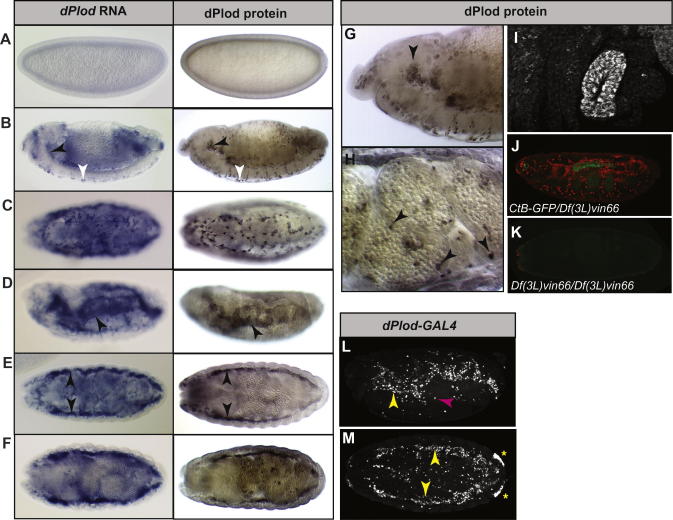
Embryonic dPlod expression. A-F, RNA *in situ* hybridisation (left panel) and dPlod antibody staining (right panel) in *Drosophila* embryos. (A) Cellular blastoderm, showing that dPlod is not found in the early embryo. (B) Stage 13 embryo, dPlod is detected at high levels in haemocytes throughout the embryo. Haemocytes in the head (black arrowheads) and adjacent to the CNS (white arrowheads) are indicated. (C) Ventral view of a stage 15 embryo showing dPlod expressing haemocytes outlining the CNS. (D) lateral view of a stage 15 embryo showing high levels of dPlod expression in the fat body (arrowheads). (E) Dorsal view of a stage 15 embryo showing dPlod expression in the fat body (arrowheads). (F) dorsal view of stage 17 embryos. (G) Higher magnification of embryo shown in B. (H) High magnification view of haemocytes surrounding the gut in a stage 17 embryo (arrowheads). (I) High magnification view of a stage 16 embryo showing dPlod protein expression in the anal pad. J and K, Stage 16 sibling control (J) or homozygous *dPlod* deficient (K) embryos stained for dPlod (red) and GFP (green). Homozygous *dPlod* deficient embryos were identified by absence of CtB-GFP. (L) and (M), p{GAWB}NP5633 GAL4 insertion line crossed to UAS-nuclear*Lac*Z. Stage 16 embryos showing expression in fat body (yellow arrowhead), haemocytes (magenta arrowhead) and anal pads (asterisk). Lateral (L) and ventral (M) views are shown). In all figures anterior is to the left.

We performed *in situ* hybridization to determine the expression pattern of *dPlod* during embryonic development (left panel, Fig. 2). We find little or no *dPlod* in early embryos (Fig. 2A). Expression is first detected at around stage 12 in haemocytes, where it accumulates at high levels by stage 13 (Fig. 2B). At this stage *dPlod* is found in haemocytes populating the whole embryo including those in the head and those surrounding the CNS (Fig. 2B, arrowheads). *dPlod* expression in haemocytes continues throughout embryogenesis, becoming particularly high at stages 15–17. For example Fig. 2C shows high *dPlod* expression in haemocytes surrounding the CNS at stage 15. During stage 13 *dPlod* is also detected in the developing fat body and by later stages accumulates at high levels in this tissue (Fig. 2D–F, stage 15 and 17 shown).

To determine the embryonic expression pattern of the dPlod protein, we performed antibody staining using the anti-PLOD3 polyclonal antibody originally raised against human PLOD3 (Fig. 2A–E, right panel). This antibody displays significant cross-reactivity to dPlod and produces a protein expression pattern very similar to the RNA expression pattern described above. We do not find dPlod protein in early embryos (Fig. 2A). In accordance with observations made for *dPlod* RNA, dPlod protein is first detected in haemocytes during stage 12. By stage 13 dPlod is detected at high levels in haemocytes throughout the embryo (Fig. 2B). A higher magnification view of the dPlod expression in the haemocytes in the head at stage 13 and around the midgut at stage 17 are shown in Figs. 2G and H. Fat body expression of dPlod is found from stage 13. It continues to be expressed in the fat body throughout embryogenesis with very robust staining found at later stages (Fig. 2D–F). We also detect high levels of dPlod in the anal pads in late embryos (stage 16, Fig. 2I). We were unable to detect any staining in embryos homozygous for a deficiency that uncovers the *dPlod* locus, confirming the specificity of the dPlod antibody (Fig. 2K compared to sibling control embryo, Fig. 2J).

The P-element Gal4 line NP5633 (*Drosophila* Genetic Resource Centre, Kyoto) maps to the 5′ end of the *dPlod* locus. We examined the expression pattern of NP5633 to determine if it recapitulates endogenous *dPlod* expression. We find that NP5633 drives expression in haemocytes, fat body and anal pads in late embryos that closely mirrors the expression pattern we see for *dPlod* RNA and protein (Fig. 2L and M). These data further confirm the expression patterns described above and provide a useful tool that permits genetic manipulation of *dPlod*.

### 1.3 dPlod is expressed in collagen-producing cells

**Fig. 3:**
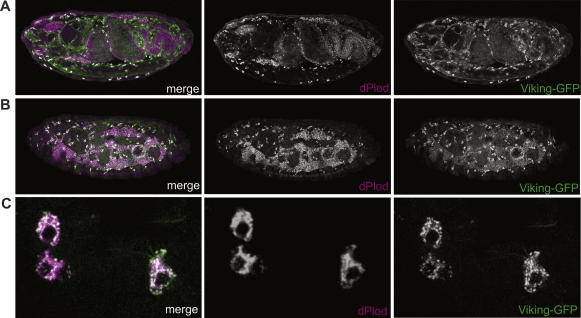
dPlod is expressed in type-IV collagen-producing cells. (A–C) Stage 16 VkgGFP embryos double stained for GFP (green) and dPlod (magenta). High levels of the dPlod protein are detected in haemocytes (A) and fat body (B) coincident with high levels of Vkg-GFP production and secretion. (C) High magnification view of haemocytes showing partial overlap between Vkg-GFP and dPlod. Anterior is to the left, dorsal up.

In other species, LH3 is expressed and its activity is required within collagen-producing cells where it facilitates the assembly and secretion of collagens – particularly type IV and type VI collagens. There are two type IV (but no type VI) collagen genes in *Drosophila*, Viking and Cg25C, which are expressed predominantly in haemocytes and fat body (Le Parco et al., 1986a,b; Yasothornsrikul et al., 1997). Collagen secreted by these cells contributes to the basement membrane of many tissues in the embryo. To test if dPlod is expressed in collagen-producing cells in the fly, we double stained embryos for dPlod (anti-PLOD3) and for type-IV collagen, using Viking-GFP as a representative type-IV collagen (Vkg-GFP, Morin et al., 2001). Vkg-GFP is a GFP protein-trap and therefore identifies the localization of the endogenous Viking protein. We find that dPlod is co-expressed with GFP in haemocytes and fat body (Fig. 3A and B). A higher magnification view of haemocytes shows that dPlod has a perinuclear localisation that partially overlaps with intracellular Vkg-GFP (Fig. 3C). The high level of dPlod in collagen-producing cells is consistent with previous observations made in other species, and with its predicted function as a collagen biosynthesis enzyme.

### 1.4 Subcellular localization of dPlod

**Fig. 4:**
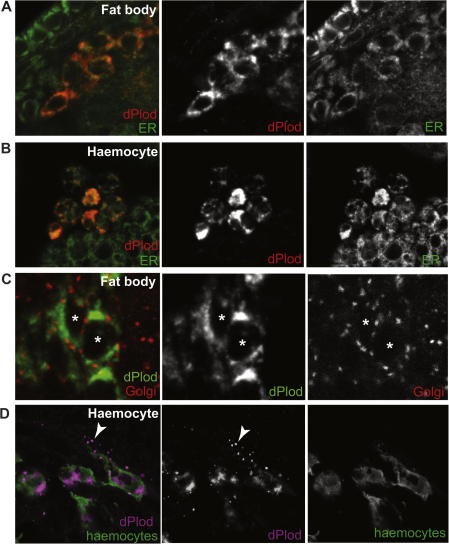
Subcellular localisation of dPlod. (A) and (B) dPlod localises to the ER. dPlod (red) in the fat body (A) and haemocytes (B) is coincident with an ER marker (EYFP-ER, green). (C) In contrast there is no overlap between dPlod (green) and a Golgi apparatus marker (EYFP-Golgi, red, overlap would appear yellow. Image from fat body shown; two individual fat body cells are marked with asterisks). (D) dPlod (magenta) expression in haemocytes (srp-Gal4, crq-Gal4, UAS-GFP; green) from a stage 16 embryo. dPlod is detected within haemocytes and extracellular puncta in areas surrounding haemocytes (arrowheads).

In mice LH3 protein is located intracellularly within the endoplasmic reticulum (ER) and it is also secreted and found in the extracellular space (Salo et al., 2006a,b; Wang et al., 2009). We examined the subcellular localization of dPlod in the fat body and haemocytes at high resolution. We find that dPlod localization overlaps substantially with a marker for the ER (EYFP-ER, (LaJeunesse et al., 2004) in fat body cells and haemocytes (Fig. 4A and B). Localization of dPlod is consistent with its possession of a putative ER-retention signal (Fig. 1A). In contrast, double staining for dPlod with a Golgi specific marker (EYFP-Golgi, (LaJeunesse et al., 2004) demonstrates that dPlod is absent from this cellular compartment (Fig. 4C). In addition, examination of haemocytes at high magnification revealed dPlod-positive staining in small puncta in the extracellular space surrounding haemocytes (Fig. 4D). This staining was not exclusively found in the extracellular space surrounding haemocytes but was more concentrated here than in haemocyte-free regions within the body cavity (data not shown). These observations suggest that dPlod, similar to mouse LH3 (Salo et al., 2006a,b; Wang et al., 2009), is secreted into the extracellular space and may act to further modify collagen within this compartment.

### 1.5 dPlod is required for type-IV collagen secretion

**Fig. 5:**
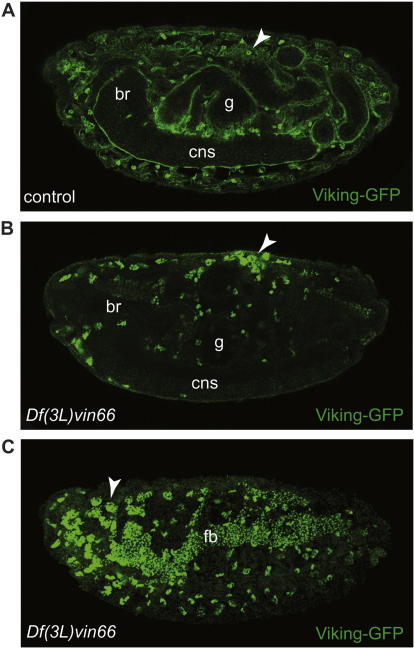
dPlod is required for type-IV collagen secretion. (A) Stage 16 Vkg-GFP control embryo showing secreted Vkg-GFP in the basement membranes ensheathing most tissues including the gut (g), central nervous system (cns) and brain (br). Vkg-GFP expression is also detected in haemocytes (arrowheads). (B) and (C), Two different focal plane images from a stage 16 *Df*(*3R*)*vin66* mutant embryo. Vkg-GFP is not secreted and is absent from the basement membranes surrounding internal tissues (B), despite its expression and accumulation to high levels in haemocytes (arrowhead) and fat body (fb) (C). Equivalent confocal settings were used to acquire images in (A–C). Anterior to the left, dorsal up.

LH3 activity is critical for the biosynthesis of type-IV collagen in humans, mice, fish and worms (Schneider and Granato, 2006; Norman and Moerman, 2000; Rautavuoma et al., 2004). In the absence of LH3, type-IV collagen is expressed but is retained within the type-IV collagen-producing cells, indicating that processing and/or secretion of type-IV collagen is disrupted. As a consequence, type-IV collagen is not incorporated into basement membranes leading to severe defects in their assembly. To determine if dPlod has a similar function we examined type-IV collagen distribution (using Vkg-GFP) in embryos lacking dPlod using a deficiency chromosome that removes the *dPlod* locus (and eight additional genes). In wild-type stage 16 embryos Vkg-GFP is detected intracellularly in haemocytes and fat body and in the basement membranes surrounding most tissues including the central nervous system (CNS) and gut (Fig. 5A). Vkg-GFP in the basement membranes is derived largely from Vkg-GFP produced and secreted from haemocytes and fat body (Yasothornsrikul et al., 1997). In stage 16 *dPlod* deficient embryos Vkg-GFP is detected in haemocytes and fat body, where it accumulates at high levels, but in contrast to wild-type embryos it is not secreted and is therefore absent from the basement membrane of the CNS, gut and all other tissues (Fig. 5B and C). Consistent with other collagen-modifying enzymes (e.g. SPARC, Martinek et al., 2008), *dPlod* homozygous deficient animals die as embryos highlighting the importance of type-IV collagen secretion for normal completion of embryonic development. Whilst we cannot rule out a role played by the other eight genes removed by the deficiency, this experiment strongly suggests that dPlod plays a critical and evolutionarily conserved role in the secretion of type-IV collagen in the fly.

### 1.6 Discussion

We have provided the first comprehensive description of the only lysyl hydroxylase encoded in the fly genome and characterised its embryonic expression pattern in detail. The dPlod protein shares a high degree of sequence identity/similarity with LH3 proteins from widely separated animal phyla with strongest conservation occurring in the catalytic domains. This suggests that fly LH3, like its counterparts in other species, possesses (i) lysyl hdroxylase, (ii) glucosyltransferase, and (iii) galactosyltransferase activities. Biochemical analysis of dPlod will be necessary to establish these predicted enzymatic activities.

Consistent with its predicted function as a collagen-modifying enzyme dPlod is highly expressed in type-IV collagen-producing cells particularly haemocytes and fat body, where it localises to the ER and partially overlaps with intracellular type-IV collagen. This is in agreement with previous reports demonstrating that vertebrate LH3 localises to the ER. Interestingly, we also detect dPlod in the extracellular space raising the possibility that dPlod modifies extracellular collagen. There is evidence that mouse LH3 is secreted, and required extracellularly for normal basement membrane structure (Salo et al., 2006b; Wang et al., 2009). These extracellular function(s) of LH3 are not well characterised, and the fly could provide a useful model to examine mechanisms of LH3 secretion and extracellular activity.

Using a genetic deficiency that removes *dPlod* we show that dPlod activity is required in haemocytes and fat body to facilitate collagen secretion. This function for dPlod is consistent with LH3 functions in other species from worms to mammals, indicating that collagen processing by LH3 enzymes is an ancient and widely conserved feature within the animal kingdom. LH3 depletion result in diverse developmental phenotypes whose unifying feature can be traced to abnormalities in the basement membrane. These phenotypes include defects in zebrafish motor neuron migration (Schneider and Granato, 2006); defects in tissue integrity in the worm (Norman and Moerman, 2000); and global defects in basement membrane assembly that correlate with general developmental retardation and result in embryonic lethality in the mouse (Rautavuoma et al., 2004; Risteli et al., 2009; Ruotsalainen et al., 2006). In humans, mutations in the LH3 gene result in a severe connective tissue defects with features that overlap with collagen disorders. Although the underlying causes of this condition have not been examined in detail, it is likely that defects in the basement membrane contribute to the disease phenotype. In the fly, mutations in *dPlod* are likely to have pleiotropic phenotypes that overlap with those in collagen IV mutants or in mutations in other genes required for collagen biosynthesis (Martinek et al., 2008). In support of this, we have observed that renal tubule morphogenesis, a processes dependent upon a haemocyte-secreted collagen sheath surrounding the tubule, is disrupted in the absence of dPlod and that these defects are similar to those for type-IV collagen mutants or for mutants in collagen processing enzymes such as dSPARC (Bunt et al., submitted for publication; (Martinek et al., 2008). This study establishes the fly as a genetically tractable model for the study of LH3 function in development and disease.

## 2 Experimental procedures

### 2.1 *in situ* hybridisation and immunohistochemistry

For *in situ* hybridisation the following primer pairs (Sigma–Genosys) were used to generate a DNA template from genomic DNA using standard PCR conditions: 5′-GGC AGA CGC CAT TAA AAG TCC-3′ and 5′-CGC TGG ATG AGA TCT TTG AGA-3′. A sense strand probe was used as a negative control. Hybridisations were carried out using standard protocols (Nagaso et al., 2001; Tautz and Pfeifle, 1989). For PLOD antibody staining, embryos were fixed in 8% formaldehyde and used fresh. PLOD signal was amplified using a biotinylated secondary antibody followed ABC (vectorlabs) and Tyramide (Perkin–Elmer) amplification kits. The following antibodies were used: rabbit anti-PLOD (ProteinTech Group, 1:50); goat anti-GFP (Abcam, 1:500); rabbit anti-Bgal (Cappel, 1:1000).

### 2.2 Fly stocks

*srp-Gal4, crq-Gal4, UAS-GFP* (gift from Will Wood) was used to visualise haemocytes. Viking-GFP (Morin et al., 2001) was used to visualise endogenous Vkg protein; EYFP-ER and EYFP-Golgi (Bloomington stock centre) were used for ER and Golgi apparatus markers. *Df*(*3L*)*vin66* carried on the TM3 chromosome (Bloomington stock centre) was used to remove the *dPlod* locus. This deficiency removes eight genes in addition to *dPlod*. The GAL4 insertion line used was p{GAWB}NP5633 (Kyoto stock number 105045).
